# Enhancing Bone Repair: Impact of Raloxifene-Functionalized Cerabone^®^ on Rat Calvarial Defects

**DOI:** 10.3390/jfb16020059

**Published:** 2025-02-11

**Authors:** Laura Gabriela Macedo, Gabriel Mulinari-Santos, Natália Barbosa de Siqueira, Letícia Pitol-Palin, Ana Cláudia Ervolino da Silva, Paula Buzo Frigério, Paulo Roberto Botacin, Paulo Noronha Lisboa-Filho, Roberta Okamoto

**Affiliations:** 1 Department of Basic Sciences, Araçatuba School of Dentistry, São Paulo State University “Júlio de Mesquita Filho”, Araçatuba 16018-805, SP, Brazil; lauragmacedo@unesp.br (L.G.M.); paulo.botacin@unesp.br (P.R.B.); 2 Department of Diagnosis and Surgery, Araçatuba School of Dentistry, São Paulo State University “Júlio de Mesquita Filho”, Araçatuba 16015-050, SP, Brazil; natalia_siqueira_@hotmail.com (N.B.d.S.); leticia.p.palin@unesp.br (L.P.-P.); ana.ervolino@unesp.br (A.C.E.d.S.); paula.frigerio@unesp.br (P.B.F.); 3 Department of Physics, Bauru School of Sciences, São Paulo State University “Júlio de Mesquita Filho”, Bauru 17033-360, SP, Brazil; paulo.lisboa@unesp.br

**Keywords:** bone, bone substitutes, wound healing, raloxifene

## Abstract

Bone substitutes are commonly used in bone regeneration, and their functionalization with bioactive molecules can significantly enhance bone regeneration by directly influencing bone cells. This study aimed to evaluate the potential of raloxifene-functionalized Cerabone^®^ (CB) for promoting bone repair and to highlight the implications in bone regeneration. The effectiveness of Cerabone^®^ functionalized with raloxifene via sonication or gel delivery in promoting bone repair in rat calvaria defects was assessed. Ninety-six male rats with critical-sized calvarial defects were divided into six treatment groups (n = 16): COAG (spontaneous blood clot), CB (Cerabone^®^), CBS (Cerabone^®^ sonicated alone), CBRS (Cerabone^®^ with raloxifene sonicated), CBG (Cerabone^®^ with gel vehicle), and CBRG (Cerabone^®^ with 20% raloxifene gel). After 14 and 28 days, samples were analyzed using microtomography, histomorphometry, immunohistochemistry, and fluorescence techniques. Quantitative data were statistically analyzed, comparing each group to the control CB group with significance set at *p* < 0.05. Micro-CT analysis demonstrated a significant increase in bone volume in the CBRS, CBRG, and CBS groups at 28 days compared to the CB group (*p* < 0.05). Specifically, the mean bone volume percentages for the CBRS, CBRG, CBS, and CB groups were 21.18%, 17.51%, 13.18%, and 7.8%, respectively. Histomorphometry showed increased new bone formation in the CBRS and CBRG groups at both 14 and 28 days. Fluorescence analysis revealed a significantly higher daily mineral apposition rate in the CBRS and CBRG groups at 28 days. These findings suggest that raloxifene-functionalized CB, delivered via sonication or gel, significantly enhances bone repair by improving bone volume and mineralization, highlighting its potential as an effective strategy for bone regeneration.

## 1. Introduction

Cerabone^®^ (CB), a bovine bone substitute material, has become broadly used in guided bone regeneration [1,2]. Its composition closely resembles natural bone, making it an effective scaffold for promoting osteoconduction [3] and facilitating bone reconstruction around dental implants [4]. The porous structure of CB not only provides mechanical support but also enhances vascularization and osteogenesis, critical factors for successful bone regeneration [1]. CB biocompatibility and ability to integrate with host tissue make it an alternative choice for augmenting bone volume and density in bone defects [5]. Its application in bone regeneration aims to ensure optimal conditions for new bone formation, leading to improved clinical outcomes for dental implants placement [5]. Despite its positive outcomes, CB has limitations, including its limited osteoinductivity, which does not actively stimulate new bone formation [2,6]. To overcome these questions, the functionalization of CB with platelet-rich plasma [7], hyaluronic acid and bioactive molecules [6] seeks to enhance bone cell activity, providing a scaffold for bone growth while stimulating bone formation during the regenerative process.

Raloxifene, a selective estrogen receptor modulator, is a drug widely utilized in the treatment of osteoporosis, particularly in postmenopausal women due estrogen deficiency [8]. By mimicking estrogen’s protective effects on bone, raloxifene helps not only inhibit bone resorption but mainly promotes bone formation [9]. Its action on bone occurs via the estrogen receptor stimulating of pre-osteoblastic cells via the WNT/B-catenin pathway [9]. Thus, this pharmacological action makes it an intriguing candidate for functionalization of bone grafts [10,11,12] and implants [13], since raloxifene can enhance the osteogenic properties to the biomaterial. Thus, the potential use of raloxifene in bone substitutes aims to create a composite scaffold that not only provides structural support but also actively promotes bone regeneration by osteoblast differentiation [14]. Thus, functionalizing CB with locally delivered raloxifene may enhance bone repair, addressing challenges in bone regeneration while mitigating potential systemic side effects of raloxifene, such as thromboembolism [15] and osteonecrosis of the jaw [16].

Functionalization of bone substitutes through innovative approaches can not only augment their mechanical and biological properties but also enhance their effectiveness in promoting bone regeneration [17]. Sonication has emerged as a promising technique for enhancing the incorporation of biomolecules into bone substitutes [10,18]. By using ultrasonic waves, sonication facilitates the uniform distribution of the drug within the bone substitute matrix, improving loading efficiency and ensuring a controlled release profile [11,12]. On the other hand, the use of hydroxy cellulose gels has also been explored for the delivery and incorporation of active biomolecules in bone repair [19,20], due to its biocompatibility and scaffold properties [21,22]. Thus, sonication can ensure efficient loading [11], while gel application provides sustained release but less uniform distribution [20]. Therefore, this study aimed to provide insights into the potential of functionalization of CB with raloxifene via sonication or gel delivery as a method for optimizing the CB, using a well-established model of rat critical calvaria defect [20,23]. It also aims to address the lack of local raloxifene-based functionalization in bone regeneration.

## 2. Materials and Methods

### 2.1. Ethics and Design

This experimental study was approved by the Animal Ethics Committee of the University of Araçatuba, SP, Brazil, under process 0811-2019, and it was carried out in accordance with the ARRIVE 2.0 guidelines for animal research [24]. Ninety-six male rats (Rattusnorvegicus, Albinus, Wistar), weighing from 300 to 400 g, aged 3 months, were randomly divided into six experimental groups. A total of 16 animals were distributed per experimental in six experimental groups (n = 16), according to the treatment in bone defect:

COAG (spontaneous blood clot), CB (Cerabone^®^), CBS (Cerabone^®^ sonicated alone), CBRS (Cerabone^®^ and raloxifene sonicated alone), CBG (Cerabone^®^ and gel vehicle), and CBRG (Cerabone^®^ and gel of raloxifene 20%).

### 2.2. Biomaterial Preparation via Sonification

The sonification of the biomaterial followed a prior protocol [11]. For the preparation of the biomaterial, the CBS and CBRS groups underwent a sonication. The 20% proportion of raloxifene to the CBRS group was determined based on our previous studies [10,11,12]. Thus, for every 0.8 g of CB, 0.2 g of solid raloxifene was added, ensuring the homogeneous incorporation of raloxifene into the CB biomaterial, as previously validated [11]. The sonication was performed using the Sonics^®^ VCX-750 device for approximately 15 min. The sonication parameters adopted were power of 750 W; frequency of 20 kHz; and 40% of the nominal amplitude of the equipment.

To achieve a homogeneous system and reduce the particle size, ultrapure Milli-Q^®^ water was used. The ultrapure Milli-Q^®^ water allowed the cavitation bubbles to have greater solvent vapor, thus accelerating the effect of acoustic cavitation. During this process, a probe ultrasound was used, oscillating at a fixed frequency and variable potential. The rapid oscillation of this probe produced a high-energy field in the fluid. The ultrapure Milli-Q^®^ water used in this experiment served as a solvent for the solutes. Once this processing was completed, the samples were left to dry at 60 °C for a period of 8 h. A sterilization process was conducted using ultraviolet light.

### 2.3. Critical-Size Defect Creation

The critical-size defect creation was also conducted as prior study [12]. The animals were anesthetized with ketamine hydrochloride (Francotar—Virbac do Brasil Ltda., São Paulo, Brazil) associated with xylazine (Rompum—Bayer AS—Saúde Animal, São Paulo, Brazil) at dosages of 70 mg/kg and 6 mg/kg, respectively. Trichotomy was performed in the calvarial region, followed by antisepsis with Povidone-Iodine Degermant (PVPI 10%, Riodeine 10 Degermante, Rioquímica, São José do Rio Preto, Brazil).

A semilunar incision approximately 2 cm in length was made in the occipito-frontal direction using a No. 15 blade (Feather Industries Ltda, Tokyo, Japan) mounted on a No. 3 scalpel handle (Hu-Friedy, Frankfurt, Germany), followed by the complete elevation of the flap using a Molt-type dissector (Hu-Friedy, Frankfurt, Germany). Subsequently, with the aid of a 5 mm diameter trephine drill (3i Implant Innovations, Inc., Palm Beach Gardens, FL, USA) attached to a low-speed drill under abundant irrigation with 0.9% sodium chloride solution (Darrow, Rio de Janeiro, Brazil), a critical surgical defect of 5 mm in diameter was created in the parietal bone. The defect size was chosen to match a critical-sized calvarial defect, as it is a widely accepted model for evaluating bone regeneration [20,23].

After critical-size defect creation, they were filled according to the treatment groups: COAG (spontaneous blood clot), CB (Cerabone^®^), CBS (Cerabone^®^ sonicated alone), CBRS (Cerabone^®^ sonicated with raloxifene), CBG (Cerabone^®^ and gel vehicle), and CBRG (Cerabone^®^ and gel of raloxifene 20%). In the present research, CB granules (Botiss Biomaterials GmbH, Zossen, Germany) with a particle size of 0.5–1 mm were used. The hydrocellulose gel vehicle, consisting of a 2% natrosol combined with 20% raloxifene, was mixed with CB granules. A total of 0.1 mg of this gel was applied in both the CBRG and CBG groups, with the key distinction being that the gel used for the CBG group was devoid of raloxifene. The natrosol gel, a hydrocellulose-based formulation, was prepared according to protocols outlined in previous studies [19,20].

Subsequent of the bone defects filled, the soft tissues were carefully repositioned and sutured in layers using absorbable suture (polylactic acid, Vicryl 4.0, Ethicon, Johnson Prod., São José dos Campos, Brazil) in the deep layer and monofilament suture (Nylon 5.0, Mononylon, Ethicon, Johnson Prod., São José dos Campos, Brazil) with interrupted sutures in the outermost layer. In the immediate postoperative period, each animal received a single intramuscular dose of 0.2 mL of Benzathine Penicillin G (Pentabiótico Veterinário Pequeno Porte, Fort Dodge Saúde Animal Ltda., Campinas, SP, USA). The animals were kept in individual cages throughout the experiment with food and water ad libitum. Half of animals were euthanized by an anesthetic overdose of anesthesia overdose (tiopental sodium, 100 mg/kg) after the period of 14 and 28 post-operative days, respectively.

### 2.4. Processing of Calcified and Decalcified Samples

Out of the total sixteen samples per group, eight were analyzed using calcified sections for Micro-CT and fluorescence imaging, while the remaining eight were processed with decalcified sections for histological, histomorphometric, and immunohistochemical analysis. The calvarias were excised and fixed in 10% formalin for 48 h. The 14-day samples were then demineralized in 10% EDTA, dehydrated through alcohols, and processed in the same manner as the 28-day samples. Afterward, they were placed in xylene, embedded in paraffin, sectioned at 5 μm thickness, and stained with hematoxylin and eosin (HE) for histology and histomorphometry. For immunohistochemistry, the sections were prepared using Harris hematoxylin.

### 2.5. Micro-CT Analysis

The micro-CT analysis followed a prior study [12]. The 28-day postoperative samples, stored in 70% alcohol, were scanned using a microtomograph (SkyScan 1172; Kontich, Belgium) at the Multi-User Laboratory of the Faculty of Dentistry of Araçatuba in a horizontal position, maintaining the apico-coronal orientation. They were connected to the device’s tubes and cut to a thickness of 8.74 μm, using an X-ray set at 50 kV and 500 μA of current, following the recommendations of Lisboa-Filho et al. A 0.5 mm aluminum filter was used in a humid environment. Image capture occurred with a camera that had a pixel size of 12.45 μm, with a row count of 2672 and a column count of 4000. The images were then reconstructed using NRecon software (v1.6.9.8 SkyScan—Kontich, Belgium), applying a smoothing factor of 1, a ring artifact correction of 3, a beam hardening correction of 5%, and an image correction variation from 0.0 to 0.11. Analyses were conducted using CTAnalyzer software (CTAn v1.12.4.0; SkyScan—Kontich, Belgium). The parameters assessed included bone volume percentage (BV/TV), trabecular thickness (Tb.Th), number of trabeculae (Tb.N), and trabecular separation (Tb.Sp), as prescribed previously [25]. All analyses and data collection were conducted by a single blinded researcher (L.G.M.) who had been specifically trained for this study.

### 2.6. Histological and Histomorphometry Analysis

The histological and histomorphormetry analysis followed a prior method [23]. For that, the slides stained with HE were analyzed using an optical microscope (Leica R DMLB, Heerbrugg, Switzerland) with 4× and 40× objectives, connected to a digital image capture camera (Leica R DC 300F, Heerbrugg, Switzerland) and a Pentium III microcomputer. The digitized images were saved as JPEG files for analysis and displayed on a Samsung monitor (SyncMaster 3Ne, 15 inches). For the histological analysis, the area of bone tissue present in the central region of the bone defects was evaluated qualitatively. For the histomorphometry, the percentage of bone formed in the bone defect was measured as well as the percentage of residual biomaterial. The new bone formed was identified based on its characteristic histological features, such as the presence of osteocytes within lacunae and trabecular structures, while residual biomaterial was identified based on its distinct appearance, including its granules with lack of cellular components typically associated with bone. The data obtained from the analyses were converted from absolute pixel values to relative percentage values to minimize the impact of differences in the size of the negative. In analyzing the results, the following proportion of bone tissue was considered: New bone formed = (%; area of bone tissue/total area) × 100 and Biomaterial = (%; area of biomaterial/total area) × 100, where the total area is the sum of all considered tissues (bone tissue and connective tissue). The histomorphometry analysis and data collection were performed by a single researcher (R.O.) trained in advance for this work.

### 2.7. Immunohistochemical Analysis

The immunohistochemical processing carried out using endogenous peroxidase activity was inhibited using hydrogen peroxide, and antigen retrieval was performed using citrate buffer (pH 6.0) at 60 °C in a humid heat environment for 20 min. Endogenous biotin was blocked with skim milk for 20 min, and to prevent nonspecific staining, the antibody was prepared in a phosphate buffer solution with 1% bovine serum albumin. Primary antibodies against PECAM, RUNX-2, and Osteopontin (SC-18916, SC-390351, SC-390518; Santa Cruz Biotechnology, Dallas, TX, USA) were used to characterize the different stages of cell differentiation during the bone repair for CB, CBG, CBRG, CBS, and CBRS. The secondary antibody used was biotinylated anti-goat, produced in rabbits (Pierce Biotechnology, Waltham, MA, USA). The signal was amplified through incubation with avidin and biotin (ABC Standard Kit, Vector Laboratories, Newark, CA, USA), and the chromogen used was diaminobenzidine (Dako Laboratories). At the end of the reactions, the sections were counterstained with Harris Hematoxylin. The slides then underwent dehydration steps and were embedded in xylene, and coverslips were mounted for microscopic analysis. Negative controls by omitting primary antibodies were performed to avoid false positive analyses. An optical microscope with a 160X Leica Aristoplan (Leitz, Bensheim, Germany) objective was used, coupled with a digital image capture camera (Leica DFC 300FX, Leica Microsystems, Heerbrugg, Switzerland), connected to a Pentium III microcomputer with software for analyzing digitized images (Leica Camera Software Box, Leica Imaging Manager—IM50 Demo Software). Three images were obtained from each slide for analysis the center of the bone defect. For each of the antibodies used, the expression of these proteins was evaluated qualitatively through ordinal analysis by assigning different scores according to the number of immunolabeled cells and the area of extracellular matrix during the bone repair. Therefore, scores were created where the immunoblots were classified on a four degree scale: absent, lower, moderate, and intense, according to a previous study [23].

### 2.8. Fluorescence Analysis

Fluorescence analysis was executed according to our previous study [26]. Animals received 20 mg/kg of calcein at 14 days post-operative and 30 mg/kg of alizarin at 42 days post-operative. These fluorochromes bind to calcium during bone mineralization, allowing for visualization of bone mineralization. Samples were dehydrated in increasing concentrations of alcohol (70%, 90%, 100%), then immersed in methyl methacrylate solution. A 1% benzoyl peroxide catalyst was added to the final solution to initiate polymerization. The samples were placed in tubes, heated at 37 °C for 5 days to complete polymerization, and then sectioned longitudinally using a bench motor. After polishing with abrasive grains from 120 until 1200, the sections were reduced to 80 μm thickness and measured with a digital caliper.

The polished slices were mounted on glass slides with mineral oil and sealed to prevent leakage and dehydration. The center of the bone defect was analyzed using a Leica CTR 4000 CS SPE confocal microscope (20× Objective). Fluorescent images of calcein, which labels older bone, and alizarin, which labels new formed bone, were used to evaluate bone formation. Calcein binds to calcium in the bone matrix during its initial deposition, marking older mineralized bone, while alizarin binds to calcium in the new mineralized bone [26].

ImageJ software (Bethesda, MD, USA) was used to measure the areas of fluorochrome precipitation in μm^2^. Daily mineral apposition rate (MAR, μm/day) was calculated by measuring the distance between the outer edges of calcein and alizarin, dividing the average by the 28-day interval between fluorochrome injections in μm, following a prior study [26].

### 2.9. Statistical Analysis

The statistical analyses were conducted using GraphPad Prism 7 software (GraphPad Software; La Jolla, CA, USA). For the quantitative parameters obtained from Micro-CT (BV/TV; Tb.Th; Tb.N; Tb.S), histomorphometry (BV/TV and percentage of biomaterial), and fluorescence analysis (calcein and alizarin areas and MAR), tests for normality and homogeneity of variances were carried out to confirm the data’s distribution along the normal curve. The Shapiro–Wilk test was utilized to assess normality. Consequently, a two-way ANOVA was employed, followed by post hoc Tukey tests for the Micro-CT data, histomorphometric analysis, and fluorescence parameters. If a significant interaction effect was observed, post hoc pairwise tests were performed to compare the experimental groups with the control (CB) group. *p*-values less than 0.05 were deemed statistically significant across all analyses.

Considering an effect size of 0.5, alpha of 5%, and a test power of 80%, the sample size was determined based on histomorphometric parameters from a previous study by do Lago et al. [23], which reported Group X = 63.6% ± 2.2 and Group Y = 16.7% ± 2.5. Based on these parameters of mean and standard deviation values, a sample size of 8 animals per group was calculated for each of the two different time points (14 and 28 days). Since this prior study involved only decalcified analysis with 8 animals per group, we applied the same number of 8 for both the decalcified and calcified analyses in our study, totaling 16 animals per group, resulting in a total of 96 animals. The sample size calculation was performed using the OpenEpi online tool (http://www.openepi.com/SampleSize/SSMean.htm, version 3, open-source calculator, accessed on 6 June 2023).

## 3. Results

### 3.1. Micro-CT Results

**Figure 1:**
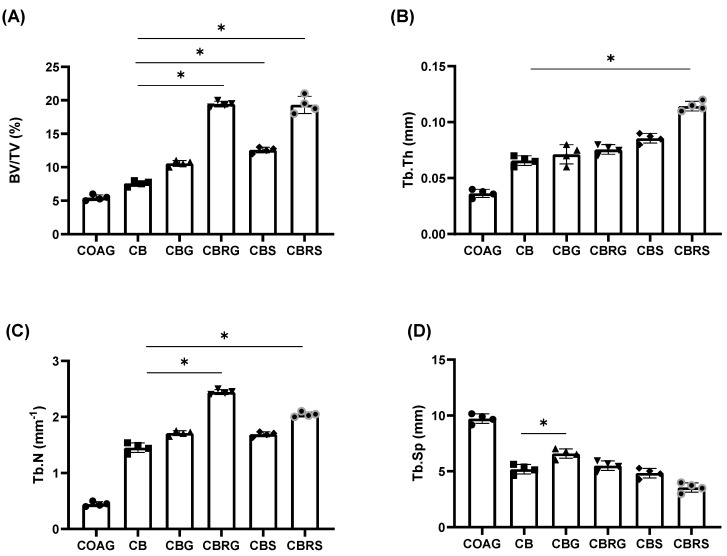
Microcomputerized tomography analysis at the bone calvarial defect. Morphologic parameters with mean results and standard deviation were calculated and reported as follows: (**A**) bone volume per tissue volume (BV/TV); (**B**) trabecular thickness (Tb.Tb); (**C**) trabecular number (Tb.N.); (**D**) trabecular separation (Tb.Sp). The * indicate a significant statistical difference (*p* < 0.05) in comparison to the CB group. Statistical tests: two-way ANOVA; Tukey post-test.

For the BV/TV parameter (Figure 1A), the best result was observed in the CBRS group with 21.18%, while the worst result was noted in the COAG group with 6.15%, followed by the CB group with 7.8%. The CBRG group obtained 17.51%, and the CBS group was 13.18%. In the case of the CBG group, it was 11.36%. There was a statistical difference between the CB group and the other groups (*p* < 0.05), except for the COAG group (*p* > 0.05).

For the Tb.Th parameter (Figure 1B), the CBRS group showed the best result (0.11 mm), while the worst result was observed in the COAG group (0.02 mm). The other experimental groups exhibited similar results; however, it was noted that the addition of raloxifene showed a slight improvement in the CBRG group. There was a statistical difference between the CB group and the CBRS group (*p* < 0.05).

For the Tb.N parameter (Figure 1C), the best result was observed in the CBRG group (2.35 mm^−1^), while the worst result was noted in the COAG group (0.4 mm^−1^). The addition of raloxifene in the CBRS group was superior to the CB group (2.01 mm^−1^ vs. 1.53 mm^−1^). Except for the COAG group, the CB group showed inferior results compared to the other experimental groups tested in this study for this parameter. There were statistically significant differences between the CB and CBRG groups (both *p* < 0.05).

**Figure 2:**
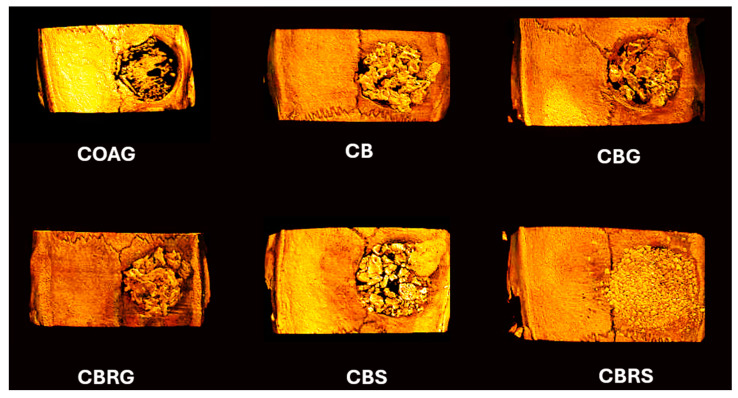
The microtomographic reconstruction of bone repair for each group at 28 days is presented. The microtomography images are representative of all six groups: COAG, CB, CBG, CBRG, CBS, and CBRS, respectively. The microtomography was performed using CTvox software (SkyScan, Version 2.7).

Lastly, regarding Tb.Sp (Figure 1D), the highest value for this parameter was observed in the COAG group (0.09 mm), while the lowest value was noted in the CBRS group (0.3 mm). It is noteworthy that the value of separation between the trabeculae was significantly higher in the CBG than in the CB group (*p* < 0.05). The representative microtomographic images of bone repair for each group at 28 days is presented in Figure 2.

### 3.2. Histological Results

**Figure 3:**
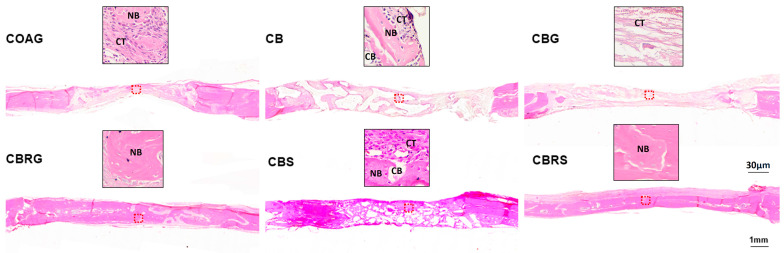
Histological images of bone repair at 14 days. The lower images of each group provide an overview in the sagittal plane, while the upper images present a detailed close-up of the tissue in the defect area for each group: COAG, CB, CBG, CBRG, CBS, and CBRS. Decalcified sections were prepared and stained with HE. NB indicates new bone formation, CT represents connective tissue, and CB denotes CB particles. The red dotted square in the lower images denotes the descriptive area from which the high-magnification areas in the upper images were derived. Scale bars represent 30 µm (upper images) and 1 mm (lower images). Original magnifications: 40× (upper images) and 4× (lower images).

**Figure 4:**
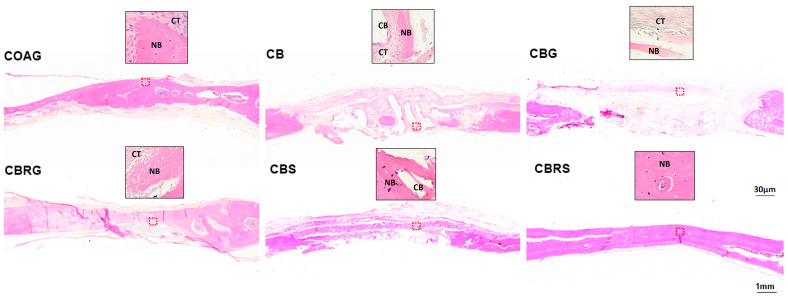
Histological images of bone repair at 28 days. The lower images of each group provide an overview in the sagittal plane, while the upper images present a detailed close-up of the tissue in the defect area for each group: COAG, CB, CBG, CBRG, CBS, and CBRS. Decalcified sections were prepared and stained with HE. NB indicates new bone formation, CT represents connective tissue, and CB denotes CB particles. The red dotted square in the lower images denotes the descriptive area from which the high-magnification areas in the upper images were derived. Scale bars represent 30 µm (upper images) and 1 mm (lower images). Original magnifications: 40× (upper images) and 4× (lower images).

The histological images of bone tissue in the central region of the defects were analyzed at 14 days (Figure 3) and 28 days (Figure 4) for the following groups: COAG, CB, CBG, CBRG, CBS, and CBRS. A detailed description of these findings is provided below.

COAG: At 14 days, the defect was filled with connective tissue containing fibroblasts in organized collagen fibers, as well as some areas of new bone formation, with no inflammatory infiltration. By 28 days, new bone formation was observed extending from the edges toward the center, though much of the defect remained filled with organized connective tissue.

CB: At 14 days, granules of biomaterial were present in the defect, surrounded by connective tissue and some areas of bone formation. By 28 days, the biomaterial remained, encased in well-organized connective tissue, with notable areas of new bone formation.

CBG: At 14 days, the defect was filled with extracellular matrix, with multinucleated cells present but no inflammatory signs. By 28 days, the biomaterial began organizing into granules with surrounding connective tissue and islands of new bone formed.

CBRG: At 14 days, the defect was filled with biomaterial and well-organized connective tissue, with islands of new bone formed. By 28 days, further organization was noted, along with an increased number of new bone formation areas.

CBS: At 14 days, homogeneous biomaterial particles were well-distributed in the defect, surrounded by cellular connective tissue and incipient bone formation. By 28 days, areas of new bone formation were evident.

CBRS: At 14 days, particles were surrounded by less cellular connective tissue and showed advanced maturation. By 28 days, smaller, homogeneous particles were observed in mature connective tissue, with bone tissue connecting the particles, indicating significant maturity in bone repair compared to other groups.

A comparison of the histological findings across the six groups is necessary to understand the impact of the different treatments on bone repair. Overall, the groups treated with functionalized raloxifene and sonicated CB, particularly CBRS, demonstrated superior bone repair and maturation. At 28 days, the CBRS group showed the most advanced bone healing, with mature bone tissue integrating with the biomaterial particles. In comparison, the CBG group exhibited the slowest bone formation, with much of the defect still occupied by connective tissue. The addition of raloxifene and sonication to CB (in the CBRG, CBRS, and CBS groups) significantly enhanced bone repair, with the CBRS group being the most effective, suggesting that the combination of these treatments plays a critical role in accelerating bone regeneration and maturation according to the histological analysis.

### 3.3. Histomorphometic Results

**Figure 5:**
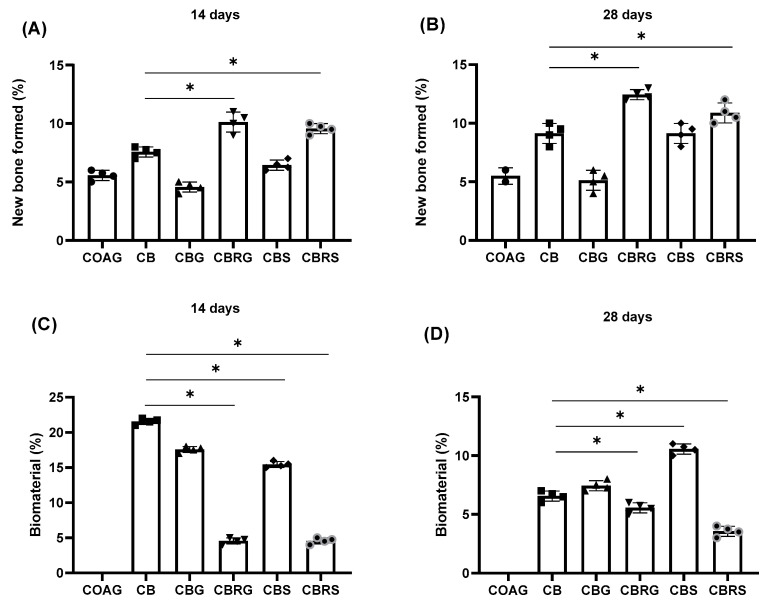
Column graphs of the histomorphometric parameters in the calvaria bone repair. Histomorphometric mean results and standard deviation of the new bone formed at 14 days (**A**) and at 28 days (**B**), following the percentage of biomaterial at 14 days (**C**) and at 28 days (**D**). The * indicates significant statistical difference in comparison to the CB group (*p* < 0.05). Statistical tests: two-way ANOVA; Tukey post-test.

For the percentage of new bone formed present in the histological slides (Figure 5), it was observed that at 14 days, the group with the highest mean value was the CBRG group with 10.05%, while the lowest mean value was in the CBG group with 4.8%. The mean of the CB group was 7.1%, for CBS was 6.78%, and for CRRS was 9.8%. At 28 days, the best groups were the CBRG and CBRS groups with 12.3% and 10.9%, and the worst value was in the CBG group with 4.96%. There was a statistical difference (*p* < 0.05) between the CB and CBRG groups and CB and CBRS groups at both 14 and 28 days.

For the percentage of residual biomaterial in the tissue, at 14 days, the CB group had the highest value, while the lowest values were found in the CBRG and CBRS groups. At 28 days, the CBS group represented the highest value, and the CBRS group had the lowest. There was a statistical difference (*p* < 0.05) at 14 and 28 days between the CB and CBRG groups, CB and CBRS groups, and CB and CBS groups.

### 3.4. Immunohistochemistry Results

**Figure 6:**
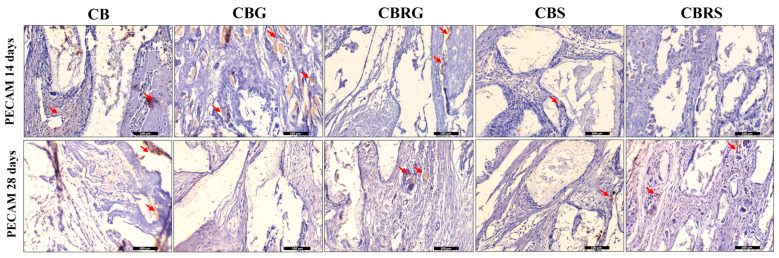
Immunohistochemical staining of PECAM at 14 and 28 days for CB, CBG, CBRG, CBS, and CBRS. The red arrows indicate the positive imunostaining for PECAM. Scale bar = 100 µm. Original magnification: 20×.

PECAM was discretely present with a lower staining in all evaluated groups (Figure 6). A possible reason for this could be the assessment periods, as 14 and 28 days may be considered late for evaluating this marker in this experimental model.

**Figure 7:**
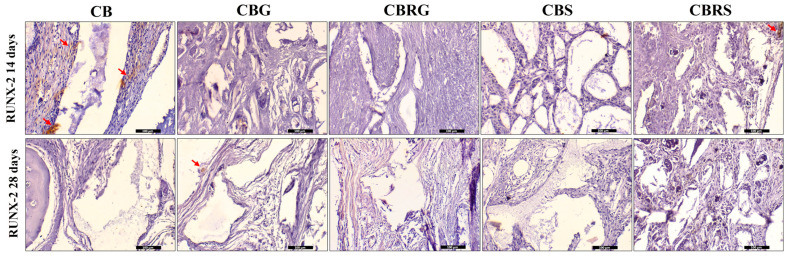
Immunohistochemical staining of RUNX-2 at 14 and 28 days for CB, CBG, CBRG, CBS, and CBRS. The red arrows indicate the positive imunostaining for RUNX-2. Scale bar = 100 µm. Original magnification: 20×.

RUNX-2, a transcription factor indicative of osteoblastic differentiation, showed moderate staining in the CB group at 14 days, while it displayed lower staining in the CBRS group at the same time point (Figure 7). At 28 days, the CBG group exhibited even lower RUNX-2 staining. In all other groups, RUNX-2 was absent at both 14 and 28 days.

**Figure 8:**
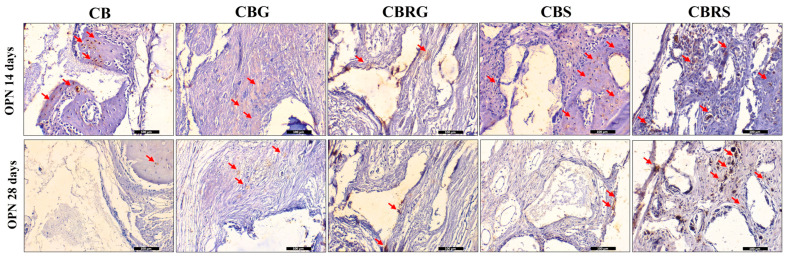
Immunohistochemical staining of osteopontin (OPN) at 14 and 28 days for CB, CBG, CBRG, CBS, and CBRS. The red arrows indicate the positive imunostaining for OPN. Scale bar = 100 µm. Original magnification: 20×.

Osteopontin showed moderate to intense staining in the CB group at 14 days (Figure 8). In the gel groups (both CBG and CBRG), staining for this protein was observed to be lower or absent. In the CBS and CBRS groups, intense staining for osteopontin was noted. In the case of the CBS group, moderate staining was observed at both 14 and 28 days, particularly near the biomaterial particles. In the CBRS group, moderate staining was also observed at 14 days and from moderate to intense at 28 days, with notable presence in the cells close the residual biomaterial.

### 3.5. Fluorescence Results

**Figure 9:**
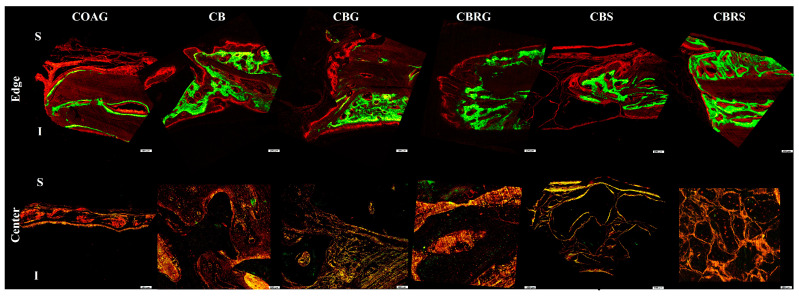
Fluorescent images showing bone mineralization at the edge (superior part) and the center of the bone defect (inferior part). Calcein (green) labels mature bone mineralization at 14 postoperative days, while alizarin (red) marks new bone mineralization at 42 postoperative days for COAG, CB, CBG, CBRG, CBS, and CBRS. The letter (S) indicates the superior part of the defect, while the letter (I) represents the inferior part of the defect. Original magnification: 20×.

**Figure 10:**
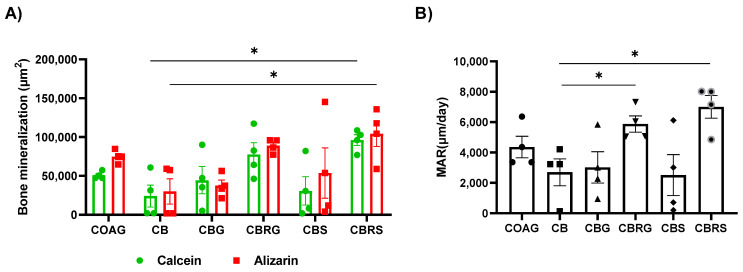
Column graphs showing bone mineralization marked by fluorochromes, with the mean and error bars. The calcein area is represented as green columns at 14 post-operative days, and the alizarin area as denoted as red columns at 42 post-operative days in μm^2^ (**A**). Daily mineral apposition rate (MAR, μm/day) during calvarial bone repair (**B**). The asterisk (*) indicates a significant statistical difference compared to the CB group (*p* < 0.05). Statistical analysis was performed using two-way ANOVA with Tukey’s post hoc test.

Fluorescent images showing bone mineralization at the edge and the center fo the bone defect (Figure 9). The areas of fluorochrome precipitation for calcein and alizarin were higher in the CBRS group compared to the CB group (Figure 10A). This suggests that the CBRS group exhibited greater mineralization compared to the CB group, as indicated by the higher areas of fluorochrome deposition.

The MAR was higher in the CBRS and CBRG groups compared to the CB group (Figure 10B). The MAR is a measure of the rate of mineral deposition, indicating how quickly new bone mineral is being laid down. This suggests that both the CBRS and CBRG groups showed enhanced bone mineralization compared to the CB group.

## 4. Discussion

Implantology has seen significant advancements with the development of bone substitutes, which are crucial for addressing bone regeneration [2]. Functionalization of these bone substitutes enhances their biological properties, improving bone graft consolidation with host tissues and overall clinical outcomes [17,27]. Thus, the effectiveness of sonification of CB functionalized with raloxifene in promoting bone repair in rat calvaria defects was tested here. The results from microtomography analysis demonstrated that CBRG, CBS, and CBRS exhibited superior results compared to conventional CB groups. Specifically, the CBRS exhibited increased Tb.Th and Tb.N in trabecular bone, indicative of improved bone quality [28]. These findings align with the higher bone mineralization MAR value from fluorescence analysis. In addition, histologic parameters reaffirmed that CBRS, as well as CBRG and CBS, yielded higher biological effects, indicating mainly that CB associated with raloxifene via sonification or gel, or even only sonicated alone had improved histomorphometry data. Notably, the CBRS and CBRG groups showed significantly better particle integration into the reparative bone, with reduced biomaterial presence at 14 and 28 days, leading to increased new bone formed.

The previous findings of studies evaluating CB as a bone substitute in bone regeneration have been promising, demonstrating its effectiveness in enhancing bone repair around dental implants even after sinus lift [29]. In preclinical models, CB has shown significant improvements in bone density and volume compared to other biomaterial controls [3]. Its porous structure facilitates not only osteoconductive but also vascularization [30], which is comparable to other bone graft integration [29,31]. Furthermore, CB has demonstrated excellent biocompatibility, fostering a favorable environment for osteoblast activity and supporting new bone formation [31]. However, strategies to enhance bone regeneration with CB are still under investigation, mainly to reduce its inflammatory response [3] and increase its osteoconductive property [11]. The findings here underscore that sonification has potential as a reliable application, particularly of CB in the incorporation and distribution of raloxifene within the biomaterial, without demonstrating higher inflammatory responses in both histological analyzed periods. In addition, both sonication groups, CBS and CBRS, exhibited strong osteopontin staining, indicating enhanced bone formation. Fluorescence data further confirmed increased mineralization, with higher MAR values in these groups.

Comparison with previous studies is essential for assessing the efficacy of raloxifene-functionalization. Our previous research investigated the combination of Bio-Oss^®^ and raloxifene 20% in maxillary sinus lift surgeries performed in rabbits, revealing enhanced cellular responses but a unexpected delayed healing associated with this combination [10]. This delay in the bone repair was not noted in the present study. Importantly, there is differences between CB and Bio-Oss in their bone reparative aspects relevant to biological responses and rate of reabsorption [32]. Here, the CB sonicated with raloxifene exhibited a greater bone formation on 14 and 28 days compared to CB. Prior studies using 20% raloxifene guided the sonication parameters in this study [11,12]. The 20% raloxifene showed improved bone volume compared to the 10% raloxifene with Bioglass^®^ in rat calvarial defects [12]. Additionally, CB with 20% raloxifene yielded better results than pure CB or sonicated CB in rabbits after sinus lift [11]. However, raloxifene-functionalized CB via sonification and raloxifene in natrosol hydrocellulose gel had not been tested in rat calvarial defects. Surprisingly, this gel-based delivery of raloxifene also yielded improved bone repair. This finding highlights the potential of using gel-based delivery for raloxifene. Natrosol hydrocellulose gel demonstrated enhanced bone formation when used to deliver doxycycline alone [19] or when associated with autologous bone graft [20], both in rat calvaria defects.

Contrast with different experiments highlights unique strategies for enhancing bone regeneration through the functionalization of bone substitutes. A prior study using a nano-hydroxyapatite substitute functionalized with bone morphogenetic protein-2 and zoledronic acid showed significant improvements in healing rat calvarial defects of 8.5 mm, achieving a bone volume of 15 mm^3^ after 8 weeks, as observed by Micro-CT data [33]. In our study, CB functionalized with sonicated raloxifene demonstrated an even greater bone volume of 21.18% after 28 days, indicating superior bone formation compared to the 8-week timeline in the first study. Another study using local melatonin combined with Bio-Oss^®^ showed a bone volume of 9.74 mm^3^ after 30 days in similar 5 mm defects, with improvements in bone healing but no significant differences in trabecular thickness or bone formation compared to Bio-Oss^®^ alone [34]. In contrast, our study showed that raloxifene-functionalized CB outperformed CB alone in both bone volume and trabecular thickness. These findings suggest that raloxifene enhances bone repair by promoting mineralization and increasing trabecular thickness, making it an effective strategy for improving bone quality in bone regeneration. However, it is important to note that the amount of biomaterial and their properties, as well as the different experimental conditions of the cited studies, can introduce variability in the results.

The incorporation of raloxifene as a functionalizing agent has garnered attention due to its positive effects on bone in previous results [10,14]. In our preceding outcomes, we also demonstrated that systemically raloxifene improved bone repair by reversing impaired osseointegration in osteoporotic rats, likely through the modulation of the RANKL/OPG ratio [35]. Moreover, other studies have shown raloxifene’s ability to inhibit osteoclast activity, while promoting osteoblast function can significantly enhance the osteogenic properties of bone substitutes [10] and implants [35,36]. Based on fluorescence data, enhanced and faster bone mineralization provided by raloxifene may offer greater bone support for implantation, potentially improving the success of bone grafts or implants in clinical settings. In this sense, another study revealed that raloxifene-functionalized iron oxide nanoparticles can lead to increased mineralization and accelerated bone fracture repair, besides anti-bacterial activity [37]. Thus, the functionalization of bone substitutes with raloxifene could be tested in the context of peri-implantitis. It can be tested even to treat and prevent infections around bone and implants, as the actual proposes of bone substitutes [27]. The integration of raloxifene into the CB group may thus offer a multifaceted approach to improving outcomes in implantology. Fortunately, the local use of raloxifene also can reduce its side effects as thromboembolism events previously reported [15].

The different analysis and limitations of this study should be explored. Herein, it is valuable to consider the different results between Micro-CT and histomorphometry data. In Micro-CT, the result is obtained from a tridimensional analysis in the bone defect. In other words, the mineralized bone in the bone defect was completely measured. On the other hand, the histomorphometry data were measured from a histological section in the middle of the bone defect parallel to the sagittal plan of the rat skull. For this reason, the results from both analyses were partly different. In Micro-CT, CBRS and CBRG have been shown with higher BV/TV values, and in the histomorphometry, it was CBRG and CBRS, respectively. By inspecting the Micro-CT data, the bone microarchitecture can be visualized, revealing that the highest Tb.Th was achieved in the CBRS group. Additionally, an increased Tb.N value from the CBRG and CBRS groups also demonstrated increased bone quality. In contrast, CBG has demonstrated a decrease in the bone quality according to the higher Tb.Sp values. Interestingly, histomorphometric analysis exposed that the incorporation of raloxifene into CB accelerated the biodegradation of CB in the CBRG and CBRS groups, in proportion to the amount of biomaterial. Supporting our data, similar findings have been previously reported, where the incorporation of raloxifene accelerated allograft bone resorption but also promoted new bone formation.

Based on the outcomes, sonication demonstrates an novel technique to enhance the functionalization of CB, improving drug incorporation, as previously reported [13,17]. Sonification allows a proportional incorporation of raloxifene within the CB matrix [11], ensuring that the drug was effectively loaded for action. This probably made the results of raloxifene with sonification in the CBRS partially better in terms of the staining of ostepontin, trabecular thickness, bone mineralization, and MAR value. Sonication may play a crucial role in enhancing the bioavailability of bone grafts by improving the distribution of bioactive drugs, leading to better outcomes, even when using gel-based raloxifene, where the distribution tends to be irregular [20]. This enhanced distribution likely contributes to the superior results observed in the CBS group, being even more pronounced in the CBRS group with raloxifene. Thus, these findings suggest that raloxifene-functionalized CB, whether delivered via sonication or in gel, could be a strategy for developing bone substitutes with promising clinical applications. While raloxifene is typically administered systemically, both delivery methods here utilize local delivery. These local approaches may reduce potential systemic side effects, like osteonecrosis of the jaw [16], while favoring bone healing and mineralization. It could also be tested as a targeted treatment for osteoporosis and may be particularly beneficial for patients who cannot tolerate oral raloxifene. However, future studies are still needed to evaluate the mechanical properties, pharmacokinetics, release profile, and toxicity of the raloxifene-loaded system.

## 5. Conclusions

Despite the limitations of this preclinical study, raloxifene-functionalized CB, delivered via sonication or gel, significantly enhances bone repair in rat calvaria defects by increasing bone volume and trabecular number, as well as enhancing bone mineralization. Therefore, the functionalization of CB with raloxifene is a promising strategy for bone regeneration. Further research is needed to evaluate the mechanical properties and pharmacokinetics of the raloxifene-loaded delivery system.

## Data Availability

The original contributions presented in this study are included in the article. Further inquiries can be directed to the corresponding authors.
